# Quantum correlation-enhanced dual-comb spectroscopy

**DOI:** 10.1038/s41377-025-01891-1

**Published:** 2025-08-01

**Authors:** Zhuoren Wan, Yuan Chen, Xiuxiu Zhang, Ming Yan, Heping Zeng

**Affiliations:** 1 State Key Laboratory of Precision Spectroscopy, and Hainan Institute, East China Normal University, Shanghai, China; 2 Chongqing Key Laboratory of Precision Optics, Chongqing Institute of East China Normal University, 401120 Chongqing, China; 3 Hainan Institute of East China Normal University, 572025 Sanya, China; 4 Jinan Institute of Quantum Technology, 250101 Jinan, Shandong China

**Keywords:** Infrared spectroscopy, Frequency combs, Mid-infrared photonics

## Abstract

Dual-comb spectroscopy (DCS) is a powerful technique for spectroscopic sensing, offering exceptional spectral bandwidth, resolution, precision, and speed. However, its performance is fundamentally limited by quantum noise inherent to coherent-state optical combs. Here, we overcome this barrier by introducing quantum correlation-enhanced DCS using correlated twin combs generated via seeded four-wave mixing. One comb acts as a local oscillator to decode molecular signals, while the twin suppresses shot noise through intensity-difference squeezing, achieving a 2 dB signal-to-noise ratio improvement beyond the shot-noise limit—equivalent to a 2.6× measurement speed enhancement. Notably, when coupled with up-conversion spectroscopy, our technique records comb-line-resolved, high-resolution (7.5 pm) spectra in the critical 3 μm region for molecular fingerprinting. These results bridge quantum optics and frequency comb spectroscopy, offering great potential for trace gas detection, precision metrology, and chemical analysis. Future developments in detector efficiency and nanophotonic integration could further enhance its scalability and impact.

## Abstract (web summary)

Quantum correlation-enhanced dual-comb spectroscopy improves signal-to-noise ratio by 2 dB beyond the quantum noise limit, offering great potential for sensitive trace gas detection, precision metrology, and chemical analysis.

## Graphical abstract

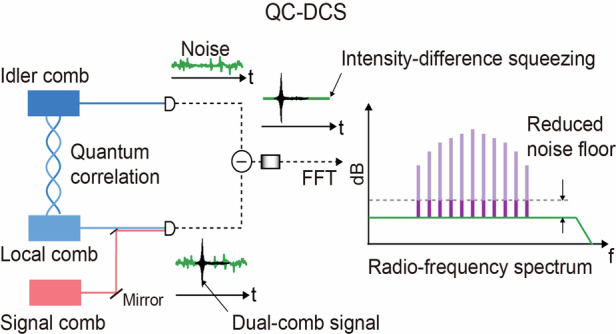

## Introduction

Spectroscopy is a cornerstone of modern science, with applications ranging from atmospheric monitoring to probing the fundamental constants of nature. Traditional techniques, while powerful, often struggle with spectral width, resolution, and acquisition speed, limiting their utility in time-sensitive or precision-demanding applications. Dual-comb spectroscopy (DCS) has revolutionized this landscape^1,2^. By leveraging the interference of two optical combs, it directly maps spectral information onto the radio-frequency (RF) domain, enabling rapid, broadband, and high-resolution measurements without the use of moving parts.

DCS has driven advances in both fundamental and applied sciences, from probing molecular dynamics^3,4^ to enhancing remote sensing technologies^5–7^. Its potential for gas sensing becomes even greater when extended to the mid-infrared region (2–20 μm^8–13^), where molecular absorptions are strong. Direct mid-infrared detection is, however, limited by high detector noise, far exceeding that of near-infrared detectors. Alternatively, up-conversion techniques^14,15^, which shift mid-infrared light to the near-infrared or visible regions for detection, have enabled mid-infrared spectroscopy and imaging, even with single-photon sensitivity^16^. Yet, in the near-infrared regime^17,18^, the performance of DCS remains constrained by the shot-noise limit imposed by coherent-state optical combs, especially in low-signal environments. Breaking this barrier could unlock new potential in molecular spectroscopy^19^ and gas sensing applications^20^.

Quantum techniques, such as squeezing and entanglement, offer pathways to surpass classical sensitivity limits, as demonstrated in gravitational wave detection^21^ and biomedical imaging^22^. Despite their promise in spectroscopy^23–26^, integrating these methods into DCS remains challenging, particularly in generating and maintaining quantum states within dual-comb architectures. While entanglement-enhanced DCS is predicted to offer 10 dB of quantum advantage within practical power ranges (µW–mW)^27^, experimental validation remains absent. Recently, progress has been made in quadrature-squeezed DCS with nearly 3 dB of metrological improvement for broadband molecular fingerprinting^28^, though its utility is restricted to the near-infrared. As quantum-enhanced DCS is still in its infancy, it requires solutions that leverage quantum techniques while preserving the strengths of DCS, including its accessibility in the mid-infrared.

Intensity-difference squeezing (IDS), leveraging quantum correlations between two optical fields to suppress intensity noise below the shot-noise limit, holds significant promise. It can be generated via various nonlinear media, such as atomic ensembles^29^ and fibers^30–32^, with atomic platforms yielding high squeezing levels (e.g., >10 dB^33^) and fibers enabling broad spectral coverage. IDS has advanced continuous-wave (CW) laser-based interferometry^34^, enabling weak signal detection and tests of fundamental physics^35^. These capabilities unlock new possibilities in quantum metrology^35^, sensing^36^, and imaging^37^.

Here, we demonstrate quantum correlation-enhanced DCS (QC-DCS) by integrating IDS into a dual-comb interferometer. QC-DCS employs bright, correlated twin combs: one serves as a local oscillator to amplify a weak signal comb encoding molecular fingerprints, while the other balances shot noise. By shielding squeezed combs from direct interactions with molecules, this innovation offers two key advantages over the previous demonstrations: (1) practicability for measurements under strong absorption (or attenuation) and (2) compatibility for integration with diverse spectroscopic platforms. As a proof-of-concept, we demonstrate QC-DCS in the context of up-conversion mid-infrared spectroscopy. As a result, we achieve, for the first time, squeezed dual-comb spectra with well-resolved comb lines in the mid-infrared, enabling multiplexed, high-resolution molecular fingerprinting. Our method not only mitigates noise limitations in DCS but also establishes a new paradigm for quantum-enhanced spectroscopy.

## Results

### Basic principles

**Fig. 1:**
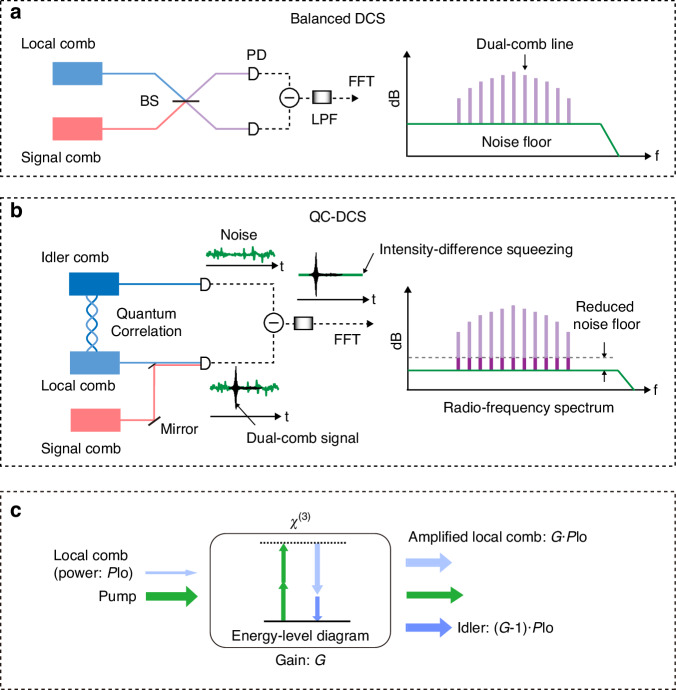
Schematics of quantum correlation-enhanced DCS (QC-DCS). **a** Balanced dual-comb spectroscopy (DCS) scheme. **b** DCS with intensity-correlated combs. **c** Seeded degenerate four-wave mixing. In **a**, a beamsplitter (BS) combines the signal and local combs for balanced detection, minimizing comb relative intensity noise. However, this does not address comb shot noise, as it is independent and uncorrelated between the two balanced beams. In **b**, the signal and local combs mix on a photodetector (PD), generating dual-comb signals, while an idler comb is detected on a second PD to capture intensity noise correlated with the local comb. By subtracting the photocurrents, the shot noise in the dual-comb signals is partially canceled, reducing the noise floor in the spectrum obtained from a fast Fourier transform (FFT) of the signal in the time domain (*t*). A low-pass filter (LPF) further removes background signals at the combs’ repetition frequencies. In **c**, *χ*^(3)^ represents a nonlinear medium with third-order nonlinear susceptibility

In a dual-comb interferometer, the dominant noise properties are generally linked to the incident comb power (*P*)^17^, provided that the power is not large enough to reach the limit set by the detector’s dynamic range. Specifically, detector noise equivalent power (NEP) predominates in the low power regime (e.g., below a few μW^17^), while comb relative intensity noise (RIN) becomes significant at higher power levels (around the mW range). In the intermediate power range, comb shot noise takes precedence, provided that the RIN is effectively mitigated through active intensity stabilization or balanced detection (see Fig. 1a). This results in a shot noise limit (SNL), also known as coherent state SNL, for dual-comb measurements. The SNR within a detection bandwidth can then be expressed as^28^

$${{{SNR}}}\propto \frac{\sqrt{{P}_{{\rm{s}}}\cdot {P}_{{\rm{lo}}}}}{\sqrt{{P}_{{\rm{s}}}+{P}_{{\rm{lo}}}}}$$

Assuming that the signal comb power (*P*_s_) is significantly lower than the local comb power (*P*_lo_), as is typical in many sensing and ranging applications, the shot-noise-limited SNR will depend primarily on the signal comb, given by

$${{{SNR}}}\propto \sqrt{{P}_{{\rm{s}}}}$$

To enhance the SNR beyond this limit, our approach (Fig. 1b) employs two intensity-correlated combs generated in a parametric amplifier based on seeded four-wave mixing (SFWM), as shown in Fig. 1c. The parametric amplifier boosts the local comb to power of *G*·*P*_lo_, where *G* is the gain, and simultaneously generates a conjugate (idler) comb with a power of (*G*−1)·*P*_lo_. This increases the total power of both beams without altering their relative intensity difference, resulting in a two-mode squeezing state. Note that this comes at the cost of increased phase difference noise, which can be ignored because the phase is not measured, and its impact on dual-comb intensity spectra is minor^28^.

For dual-comb detection (Fig. 1b), the local comb mixes with a third comb (the signal comb) at one terminal of a balanced detector, while the correlated idler comb is directed to the other terminal to cancel the local comb shot noise via IDS. This yields an SNR of

$${{{SN}{R}}}^{{\prime} }\propto \frac{\sqrt{{P}_{{\rm{s}}}\cdot {G\cdot P}_{{\rm{lo}}}}}{\sqrt{{P}_{{\rm{s}}}+{P}_{{\rm{lo}}}}}=\sqrt{G}\cdot \sqrt{{P}_{{\rm{s}}}}$$

provided that *P*_lo_ ≫ *P*_s_ (see Supplementary Note 1). Hence, our scheme ideally improves the SNR by a factor of $\sqrt{G}$ relative to the coherent-state SNL, assuming that the primary shot noise in dual-comb measurements is quadrature-insensitive (or “intensity-like”) due to its origin in the interaction between coherent-state comb pulses and vacuum fluctuations coupled via a beam combiner. This assumption holds for dual-comb systems with small duty cycles^5–14,38^ (e.g., 10^−2^–10^−3^, also see Supplementary Fig. S1) and has been verified in the pioneering work on squeezed DCS^28^. In practice, the improvement may be reduced by optical losses during squeezing and detection^39^ and by the complex noise characteristics in DCS^17^.

Nevertheless, our work identifies a new approach to mitigating shot noise introduced by strong local comb pulses. While classical methods such as temporal gate filtering^40^ and alias spectral averaging^41^ are available, we propose a quantum correlation-based strategy that may complement these techniques (see Supplementary Note 1 for a detailed discussion). Here, we specifically choose IDS for two main reasons: (1) it potentially enables high levels of squeezing; and (2) it is phase-insensitive, avoiding the complexities of precise phase control required for quadrature squeezing.

### Quantum-correlated comb generation and characterizations

**Fig. 2:**
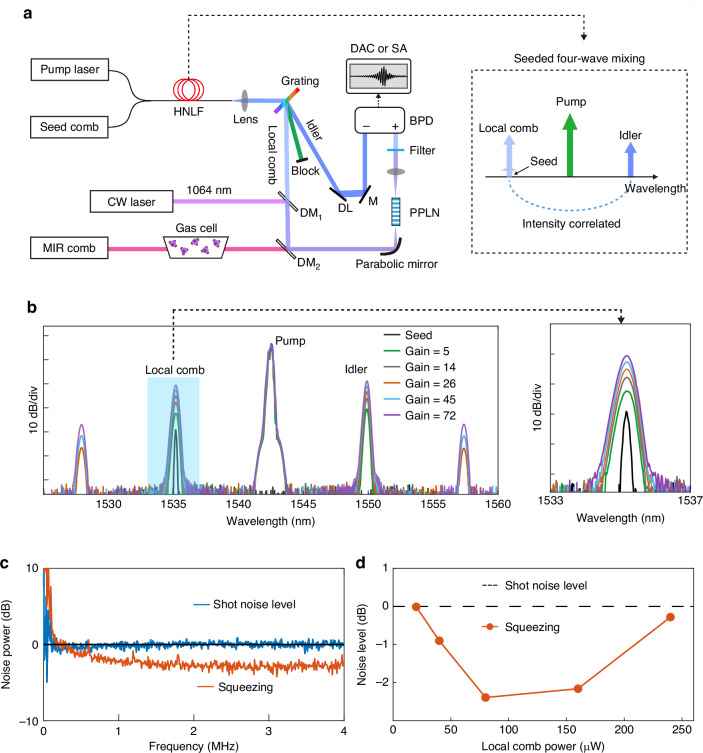
Experimental setup and characterization of intensity-correlated combs. **a** Experimental setup. MIR mid-infrared, HNLF highly nonlinear fiber, L lens, DAC data acquisition card, SA RF spectrum analyzer, BPDbalanced photodetector, DM dichroic mirrors, DL delay line, M mirror, PPLN periodically poled lithium niobate crystal. **b** HNLF output spectra at different pump powers. The spectral resolution is 0.05 nm. **c** Normalized intensity-difference noise power and the corresponding shot noise limit. The detected local comb (or idler) power is about 80 µW. The electronic noise floor has been subtracted. **d** Intensity-difference squeezing versus detected local comb power

Our experimental setup is shown in Fig. 2a (see the “Methods” section and Supplementary Fig. S2 for details). In this proof-of-concept experiment, all combs are generated using electro-optical (EO) comb generators, which exhibit excellent passive mutual coherence and broad wavelength tunability^42^. To generate quantum-correlated twin combs, we launch a seed comb (<4 µW) spectrally centering at 1535.2 nm into a piece of highly nonlinear fiber (HNLF) alongside a synchronous pump comb at 1542.5 nm. Note that both the pump and seed combs can be tuned in a wide wavelength range and examples are shown in Supplementary Fig. S3. Due to the SFWM, the HNLF amplifies the seed (local) comb and generates an idler comb at 1550 nm. The gain (*G*) of the amplification depends on the pump power. By adjusting the pump power from 32 to 40 mW, we tune the gain from 5 to 72. The spectra measured at different gains are plotted in Fig. 2b.

To verify the quantum correlations of the twin combs, we spatially separate them using a transmission grating (transmissivity >96%) and direct them to a balanced detector that automatically subtracts the photocurrents. We measure the noise power spectra of the detector output with a spectrum analyzer (10 kHz resolution bandwidth). For example, the result for *P*_lo_ = 80 µW and *G* = 20 is plotted in Fig. 2c, alongside the corresponding SNL trace. The SNL is calibrated using a coherent comb with power equal to the total power of the twin beams incident on the detector. We split this calibration comb equally with a beamsplitter for balanced detection, a method commonly used for calibrating SNL^43^. The calibration data are shown in Supplementary Fig. S4. Since the combs are pulsed, we include a time delay line in the idler beam path for temporal mode matching. This setup achieves maximum noise reduction of 3.5 dB at 3 MHz and an average of 2.3 dB from 1 to 4 MHz. This corresponds to a generated squeezing of 7.3 dB at 3 MHz (4 dB in average), when corrected for detection losses and the nonideal quantum efficiency of the detectors (Supplementary Note 2 and Supplementary Table 1). Meanwhile, the degree of squeezing deteriorates when the local comb power exceeds 80 µW or *G* ≥ 20, as shown in Fig. 2d, due to the onset of other nonlinear effects, such as cascaded FWM (e.g., signals at 1528 and 1557 nm in Fig. 2b) and stimulated Raman scattering.

In our experiment, we utilize the two correlated combs for frequency up-converted mid-infrared DCS. Specifically, we employ a mid-infrared comb generated via difference frequency generation (see the “Methods” section). After passing through a gas cell, the mid-infrared comb spatially overlaps with a 1064 nm CW laser (up to 1 W) and the local comb on a dichroic mirror. These beams are then directed to a periodically poled lithium niobate (PPLN) crystal, where the mid-infrared comb is up-converted to the near-infrared region, spectrally overlapping with the local comb. This scheme takes advantage of near-infrared detectors with a NEP below 1 pW Hz^−1/2^, which is typically unattainable with mid-infrared detectors. Innovatively, this up-conversion setup also functions as a beam combiner, minimizing losses during beam combination while preserving the quantum characteristics of the squeezed local comb to the greatest extent. The total loss introduced by this up-conversion setup is less than 0.2 dB for the local comb. Meanwhile, the up-converted near-infrared signal comb is fully utilized for dual-comb detection.

After passing through a spectral band-pass filter, the up-converted comb (signal comb) beats with the local comb at one input of the balanced detector, while the idler beam is directed to the other input. The balanced output is then digitized by a 16-bit data acquisition card (ATS9626, AlazarTech) for dual-comb measurements.

### Dual-comb measurements

**Fig. 3:**
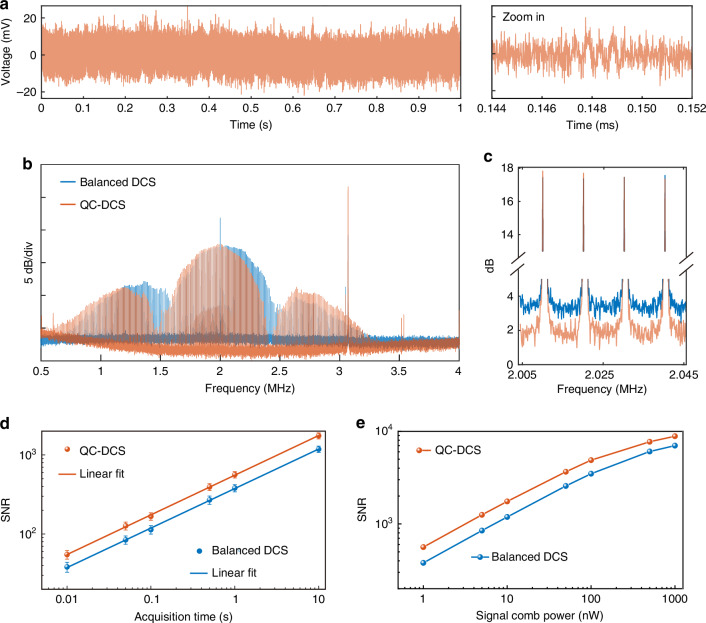
Dual-comb results. **a** Time-domain signals of QC-DCS. **b** Dual-comb spectra with resolved comb lines. **c** Zoom-in of the dual-comb lines. **d** Signal-to-noise (SNR) dependence on acquisition time. **e** SNR dependence on the signal comb power. The comb parameters are set to *f*_r_ = 203.07 MHz, Δ*f*_r_ = 10 kHz, and the offset frequency between the signal and local combs is *f*_offset_ = 2 MHz. All the data are measured in the time domain at a sample rate of 100 MS s^−1^, and then Fourier transformed into the frequency domain. In **d**, the two data sets displayed on a log-log scale are linearly fitted with an identical slope of 0.5. The SNR in **d** and **e** is calculated for the dual-comb lines in a range of 1.7–2.3 MHz with the local comb power fixed at 80 µW

Figure 3a shows a time-domain trace of QC-DCS recorded in 1 s. In this measurement, the signal comb power is only 1 nW, and the local comb is 80 µW, leading to time-domain interferometric signals that are nearly buried in noise. A comparison between squeezed interferograms and balanced dual-comb signals at the SNL, measured using two coherent-state combs under identical power conditions, is shown in Supplementary Fig. S5. IDS suppresses noise within a specific RF regime. While, in other RF regimes, the noise is not squeezed. In Fig. 3b, the squeezed RF spectrum (orange) shows a reduced noise floor from 1 to 3.5 MHz compared to a SNL dual-comb spectrum (blue). This bandwidth is limited by detector noise (see Supplementary Fig. S4). Nevertheless, we set the comb parameters properly to place all the dual-comb lines within this range. In the magnified view (Fig. 3c), the noise floor of the squeezed spectrum is 2.1 dB below the SNL, while the dual-comb line peaks remain similar. This demonstrates the advantage of our approach in enhancing SNR.

Figure 3d shows the SNR dependence on acquisition time. The SNR is defined as the ratio of dual-comb line peaks to the standard deviation (SD) of noise between adjacent lines. For both the squeezed and SNL cases, the SNR scales with the square root of acquisition time. Compared to balanced DCS, our method achieves approximately a 2 dB improvement in SNR for the same acquisition time or, equivalently, achieves the same SNR with 2.6× less measurement time, effectively a 2.6× speed-up. This benefits time-intensive measurements, such as those in remote sensing over long distances^5–7^.

Furthermore, as expected, the SNR (measured in 1 s) in both cases improves with the square root of the signal comb power (when *P*_s_ < 100 nW, as shown in Fig. 3e), indicating that shot noise dominates in the measurements. For *P*_s_ > 100 nW, however, additional noise contributions from the mid-infrared comb generation and nonlinear up-conversion processes arise, limiting the SNR. Nevertheless, our quantum-enhanced setup achieves a figure-of-merit exceeding 10^6^ Hz^1/2^,which is remarkable given the nW-level signal comb power in typical applications such as open-path dual-comb spectroscopy^6^. By comparison, state-of-the-art dual-comb systems with similar or higher figures-of-merit (10^6^–10^7^ Hz^1/2^) typically operate with incident comb powers ranging from tens of μW^14^ to tens of mW^44^.

### Spectroscopic validation

**Fig. 4:**
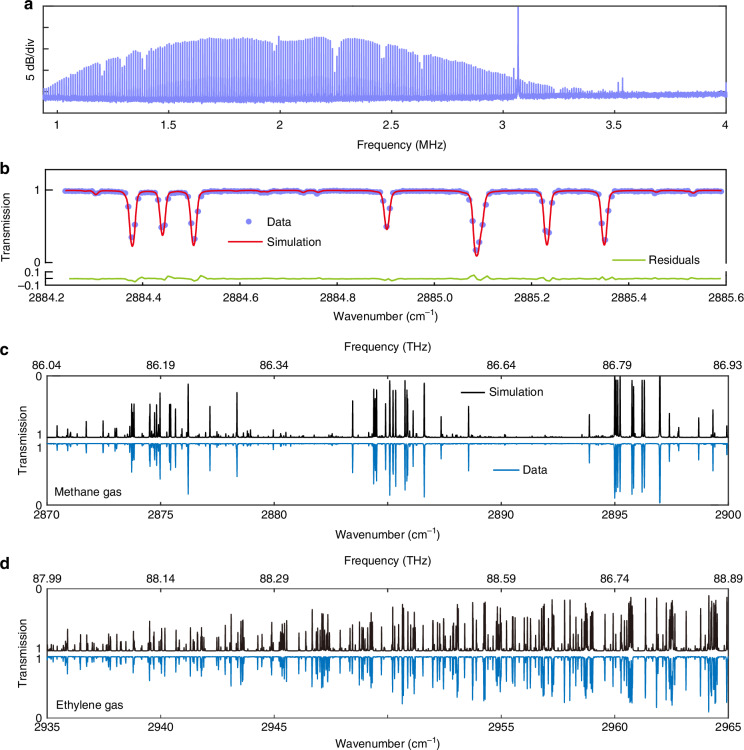
Mid-infrared molecular spectra. **a** Molecular absorption line encoded on quantum-enhanced dual-comb lines. **b** Normalized spectra. The experimental data (purple dots) are compared with simulations (red line). Their residuals are displayed in green. **c** and **d** Spectral results (blue) for CH_4_ and C_2_H_4_, respectively, together with simulation spectra (black). For the measurements, the 10-cm-long gas cell is filled with pure CH_4_ (or C_2_H_4_) at a pressure of 2000 Pa (temperature 275 K)

One key advantage of our approach is that the squeezed local comb does not interact directly with molecules, making it immune to losses from molecular absorption—a critical feature for practical gas sensing applications. In Fig. 4a, we show molecular absorption lines encoded onto the resolved dual-comb lines with reduced quantum noise. The total measurement time is 1 s, with a single-shot spectral width of approximately 40 GHz (equivalent to 1.33 cm^−1^ or 1.5 nm at 3.3 μm), covering 200 comb lines. The mid-infrared comb is spectrally tailored using an adjustable band-pass filter, producing a smooth spectral envelope. The absorption dips, revealing the fundamental transitions in the P(13) manifold of the *v*_3_ band of methane (CH_4_), cause up to 6 dB attenuation in comb line power. This level of attenuation significantly undermines quantum properties, creating substantial challenges for quantum-enhanced dual-comb schemes recently proposed^27,28^.

We validate our spectroscopic method by comparing experimental data with simulations (see the “Methods” section) using parameters from the HITRAN2020 database. Figure 4b provides an example, where the data points (purple dots) represent the dual-comb line peaks (from Fig. 4a), normalized against a background spectrum without gas. The measured data are calibrated on the wavenumber axis, using the known center wavenumber of the mid-infrared comb (see the “Methods” section) and the line spacing of 0.0067 cm^−1^ (for *f*_r_ = 203.07 MHz). The calibrated spectrum aligns well with the simulation, with residuals showing an SD of 1.8% (green curve in Fig. 4b).

Finally, we demonstrate the system’s capability for broadband spectral detection. The mid-infrared comb offers a tuning range from 3216.27 to 3602.33 nm (83.22 to 93.21 THz), covering the molecular fingerprints of many important hydrocarbon gases, including methane and ethylene (C_2_H_4_). The local comb can be tuned from 1510 to 1590 nm (188.55 to 198.54 THz), aligning with the upconverted spectral range of the mid-infrared comb. Figure 4c and d show two nomalized spectra, spanning 30 cm^−1^ or 1 THz, obtained through spectral stitching^8^, alongside corresponding simulated spectra (in black) for comparison. Those spectra reveal the transition lines in the *v*_3_ band of CH_4_ (from 86.04 to 86.93 THz) and in the *v*_11_ band of C_2_H_4_ (from 87.99 to 88.99 THz), with a spectral point spacing of 203.07 MHz (or 7.5 pm).

## Discussion

In this paper, we exploit quantum correlations in twin combs to suppress shot noise in dual-comb measurements. Our approach is implemented on an up-converted mid-infrared DCS platform, delivering quantum-enhanced, high-resolution (7.5 pm) molecular spectra in the 3 μm region. For the current setup, our method is valid within a local comb power range of 20–250 μW (Fig. 2d) and a signal power range from <1 nW to 2 μW (Fig. 3e). Nevertheless, we achieve an average of 2.3 dB of squeezing, limited by the quantum efficiency of the two detectors. With high-efficiency detectors (e.g., quantum efficiency ~98%), we predict an improvement in squeezing to 6.5 dB (see Supplementary Note 2), though such detectors are not yet accessible to us. Furthermore, exploring nanophotonic tools, such as waveguides^45^ and microresonators^46^, could significantly enhance the integration and performance of our setup. Recent advances in integrated nanophotonic chips have achieved 8 dB of broadband degenerate squeezing, observable across detection bandwidths up to 1 GHz^47^.

Meanwhile, significant progress has been made in direct mid-infrared DCS, including extended spectral coverage^9–11^ and improved SNR with balanced detection using thermoelectrically cooled HgCdTe detectors^14^. While, up-conversion detection remains promising due to ultra-low-noise, high-efficiency, fast-response near-infrared detectors and advances in high-efficiency nonlinear converters like PPLN waveguides.

Overall, we anticipate that our approach will be embraced across a wide range of applications. At its core, QC-DCS enhances frequency comb spectroscopy for weak light detection, benefiting comb-based multidimensional spectroscopy^19^ and nonlinear imaging^38^. In our method, the coherent-state signal comb used to interrogate molecules can be manipulated without compromising the degree of squeezing, enabling seamless integration with other sensitivity-enhanced platforms such as open paths^5–7^, resonance cavities^12,13,48^, multi-pass cells^3^, or hollow-core fibers^41^. Reducing shot noise in these systems can further improve sensitivity for gas sensing applications, including trace gas analysis^20^, combustion diagnostics^4^, and environmental monitoring^5–7^. This adaptability also allows our method to function effectively in complex environments, such as detecting industrial leaks or performing breath analysis for medical diagnostics^49^. Finally, by integrating quantum sources with the powerful dual-comb interferometry method, our technique has the potential to advance quantum metrology and light detection and ranging applications^50^.

## Methods

### Quantum-correlated comb generation

To produce the seed comb (Fig. 2), a wavelength-tunable CW laser (linewidth <10 kHz, 100 mW; microphotons) is input into an intensity modulator (IM) with a 40-GHz bandwidth (KY-MU-15-DQ-A, Keyang Photonics). The IM is driven by a pulse generator (Aunion Tech) that produces 30-ps electrical pulses triggered by an RF synthesizer (SMC100A, R&S). Consequently, the IM outputs 30-ps optical pulses with a repetition rate of *f*_r1_ = 203.7 MHz. After amplification in a custom-built fiber amplifier, the comb is spectrally broadened in a 100-m-long highly nonlinear fiber, achieving a full width at half maximum (FWHM) of <2 nm, depending on the comb power. The pump laser is generated in a similar manner and synchronized with the seed comb. The two are combined using a 10/90 fiber beam coupler and launched into a segment of dispersion-managed highly nonlinear fiber, where correlated twin beams are produced via seeded four-wave mixing. This fiber features a zero dispersion at 1550 nm, a linear dispersion slope of 0.03 ps nm^−2^ km^−1^, a loss coefficient of <1.5 dB km^−1^, and a nonlinear coefficient exceeding 10 W^−1^ km^−1^. To spatially separate the twin beams, a lens (*f* = 11 mm; C220TMD-C, Thorlabs) and a transmission grating (LBTEK) are employed.

### Mid-infrared comb generation and upconversion detection

The mid-infrared comb (maximum power of 5 mW at 3.3 µm) is generated via difference frequency generation between a near-infrared EO comb and a 1064 nm CW laser (optical frequency: *f*_1064_). The center wavelength and repetition frequency of the mid-infrared comb, both of which are tunable, are determined by the near-infrared EO comb. This EO comb shares the same wavelength-tunable CW laser (optical frequency: *f*_cw_) as the seed comb, ensuring mutual coherence between the two combs. The residual 1064 nm CW laser is subsequently employed for upconversion detection using a temperature-controlled, 40-mm-long periodically poled lithium niobate (PPLN) crystal (Castech) with a conversion efficiency of ~0.2 mW·W^−2^ cm.

### Calibration of optical frequencies

The center optical frequency of the dual-comb spectrum corresponds to the center frequency of the mid-infrared comb, defined as *f*_1064_−*f*_cw_, both measured with a high-precision wavemeter (AQ6150, Yokogawa; frequency uncertainty <25 MHz). This center frequency aligns with the dual-comb center at *f*_offset_ = 2 MHz, determined by two acousto-optic modulators (AOMs), one for each heterodyne comb. The AOMs facilitate shifting the dual-comb lines into the squeezed RF regime. The line spacing of the mid-infrared comb (its repetition frequency), *f*_r_ = 203.07 MHz, maps to Δ*f* = 10 kHz in the RF domain. This mapping enables the conversion of the RF axis into the optical frequency axis.

### Spectral simulation

The simulated spectra in Fig. 4 are generated using a Voigt profile, with parameters derived from the HITRAN2016 database, including molecular line strengths and line center positions for methane (CH_4_) and acetylene (C_2_H_2_). The simulation is performed under conditions of 296 K temperature, 2000 Pa pressure, a spectral resolution of 0.00751 cm^−1^, and an optical path length of 10 cm, consistent with our experimental setup.

## Supplementary information

Supplementary Information for Quantum Correlation-enhanced Dual-comb Spectroscopy

## Data Availability

The data that support the findings of this study are available from the corresponding author upon reasonable request.
