# Supplementary material for: Quantum correlation-enhanced dual-comb spectroscopy
Source: Light Sci Appl. 2025 Aug 1;14:257. doi: 10.1038/s41377-025-01891-1 (PMC12313883; doi:10.1038/s41377-025-01891-1)
Supplement: Supplementary file 1 — Supplementary Information for Quantum Correlation-enhanced Dual-comb Spectroscopy [file 41377_2025_1891_MOESM1_ESM.docx]

Supplementary Information for

**Quantum correlation-enhanced dual-comb spectroscopy**

Zhuoren Wan^1,2^, Yuan Chen^1^, Xiuxiu Zhang^1^, Ming Yan^1,2,3^*, and Heping Zeng^1,2,3,4^*

^1^State Key Laboratory of Precision Spectroscopy, and Hainan Institute, East China Normal University, Shanghai, China

^2^Chongqing Key Laboratory of Precision Optics, Chongqing Institute of East China Normal University, Chongqing 401120, China

^3^Hainan Institute of East China Normal University, Sanya 572025, China

^4^Jinan Institute of Quantum Technology, Jinan, Shandong 250101, China

*Corresponding author: *myan@lps.ecnu.edu.cn, hpzeng@phy.ecnu.edu.cn*

**This PDF file includes:**

Figures S1 to S5

Table 1

Notes 1 to 2

References 1 to 6

Supplementary Figures


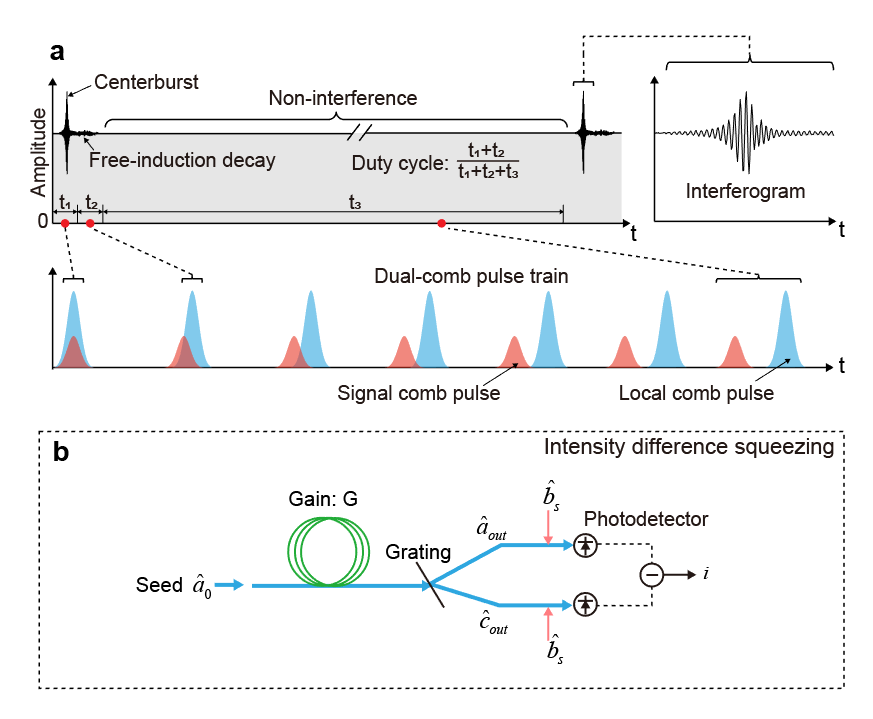


**Fig. S1** | **Basic concepts in dual-comb spectroscopy.** **a** Time-domain dual-comb interferograms. **b** Schematics of heterodyne detection with intensity difference squeezing. The operators, $\hat{a}_{0}$, $\hat{a}_{out}$, $\hat{c}_{out}$, and $\hat{b}_{s}$, represent the seed, the amplified seed, the conjugate idler, and the signal fields, respectively, and *i* denotes a photocurrent. In dual-comb spectroscopy (DCS), two combs (signal and local) beat on a single photodetector, producing interferometric signals, i.e., dual-comb interferograms, superimposed on electrical pulse trains from the combs (shown as the grey background in **a**). Since the background appears at the combs’ repetition frequencies, which are different than the interferograms, it can be removed using an electrical low-pass filter. However, this background contributes to a significant amount of relative intensity noise (RIN) and shot noise in the radio-frequency domain. Balanced detection further minimizes RIN but does not eliminate shot noise (as it is independent and uncorrelated between the two balanced beams). Recent insights into shot noise in DCS have been gained by examining its temporal properties. As depicted in **a**, the time-domain dual-comb signals over one period (t_1_+t_2_+t_3_) can be divided into three parts: an interferogram centerburst (within the time window of t_1_), a trailing free induction decay (FID) signal during t_2_, and a non-interference region (t_3_). Each of these parts exhibits distinct shot noise characteristics. In the centerburst region, the signal and local comb pulses temporally overlap, and their heterodyning on a detector yields a strong interferogram (i.e., a Fourier counterpart of the comb spectrum). This region is understood as quadrature detection of two coherent state combs. In the FID region, where the local comb pulse interacts with weak modulation signals induced by sample molecules, and in the non-interference region, where the signal and local pulses are temporally separated, the measurements are quadrature-insensitive. This can be understood as a coherent state of each comb interacting with a vacuum state at a beamsplitter. This type of shot noise may dominate in DCS for dual-comb systems with a small duty cycle (i.e., t_3_ ≫ t_1_, t_2_). This applies to spectroscopic applications like environmental monitoring and breath analysis, where spectral resolution requirements are less stringent. However, for applications such as Doppler-free molecular spectroscopy, a more advanced quantum-enhanced scheme is necessary.


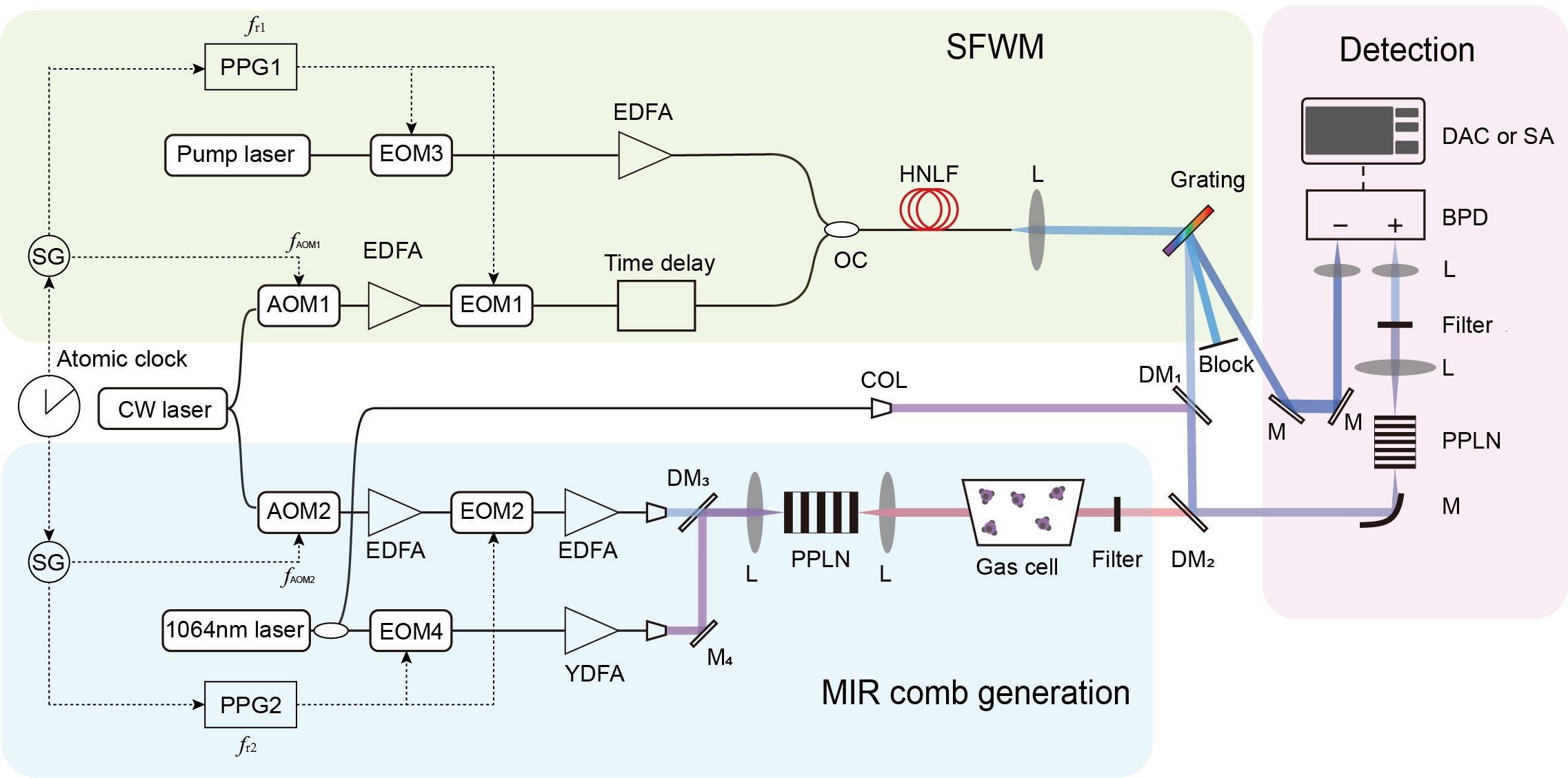


**Fig. S2** | **Experimental setup.** Abbreviations: SFWM, seeded four-wave mixing; MIR, mid-infrared; SG, radio-frequency signal generator; PPG, picosecond pulse generator; AOM 1, 2, acousto-optic modulators (driven frequency: *f*_AOM1_=80 MHz, *f*_AOM2_=82 MHz); EOM 1, 2, 3, electro-optical modulators (driven frequency: *f*_r1_=203.07 MHz, *f*_r2_= *f*_r1_ +10 kHz); EDFA, erbium-doped fiber amplifier; YDFA, ytterbium-doped fiber amplifier; HNLF, highly nonlinear fiber; COL, collimator; OC, optical coupler (50:50); L, lens; DM_1~2_: dichroic mirrors (cutoff wavelengths at 1300 nm for DM_1_ and 1800 nm for DM_2_); M, Mirror; PPLN, periodically poled lithium niobate crystal; BPD, balanced photodetector; DAC, data acquisition card; SA, radio-frequency spectral analyzer. Our setup involves a continuous-wave laser at 1064 nm and two identical wavelength-tunable lasers in the telecommunication band (labeled as “CW laser” and “pump laser”). All the electronic devices are referenced to an atomic clock with a frequency stability of 10^-13^ in 1 s.


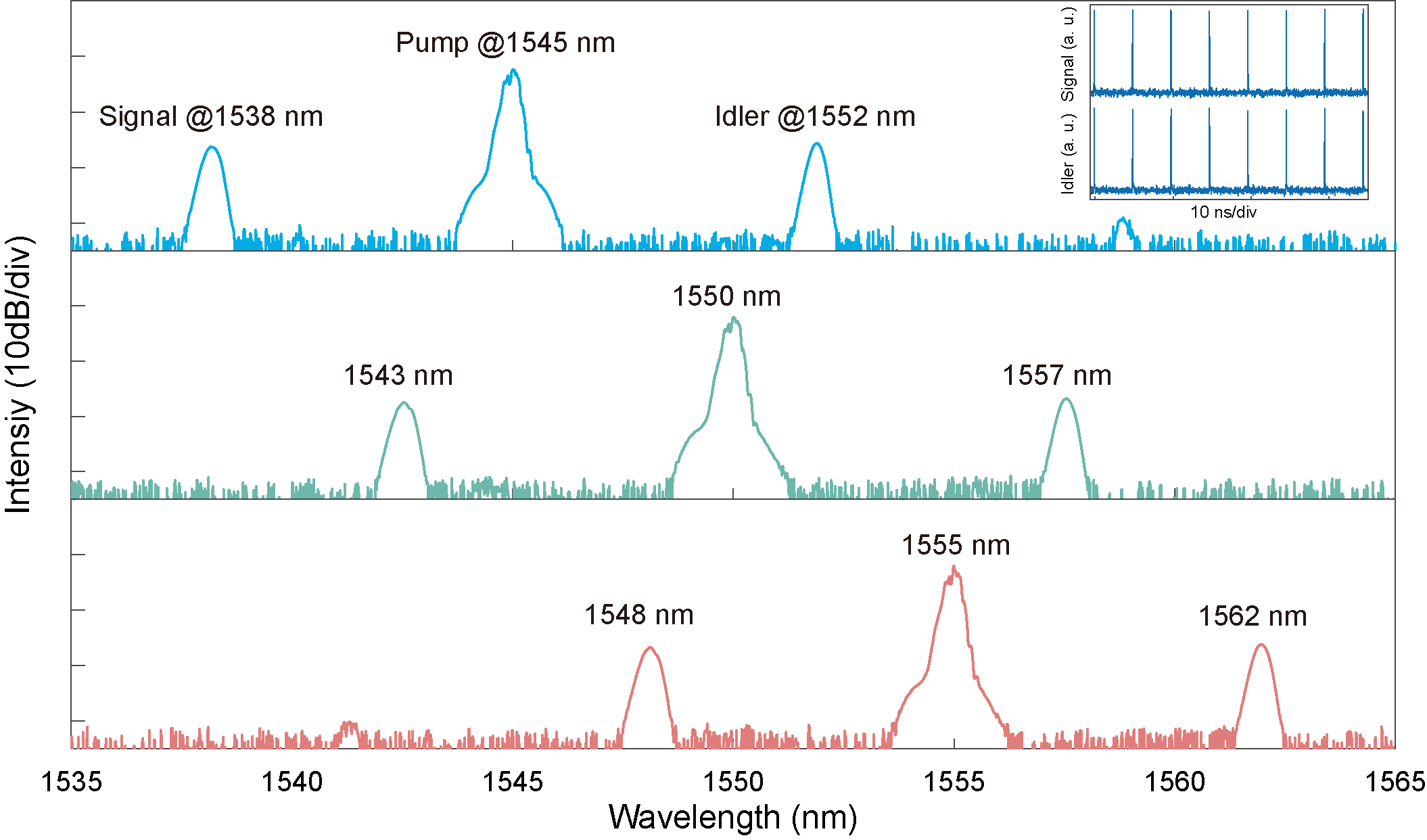


**Fig. S3** | **Spectral tuning of quantum-correlated beams.** In our setup, both the pump and the seed laser wavelengths are tunable. This tunability compensates for our combs' single-shot spectral width limitation, enabling broadband spectral detection. For the data shown here, the seed power is 4 μW (-3dB bandwidth of 0.5 nm), and the pump power is 38 mW. The inset shows the pulse trains of the seed and idler pulses.


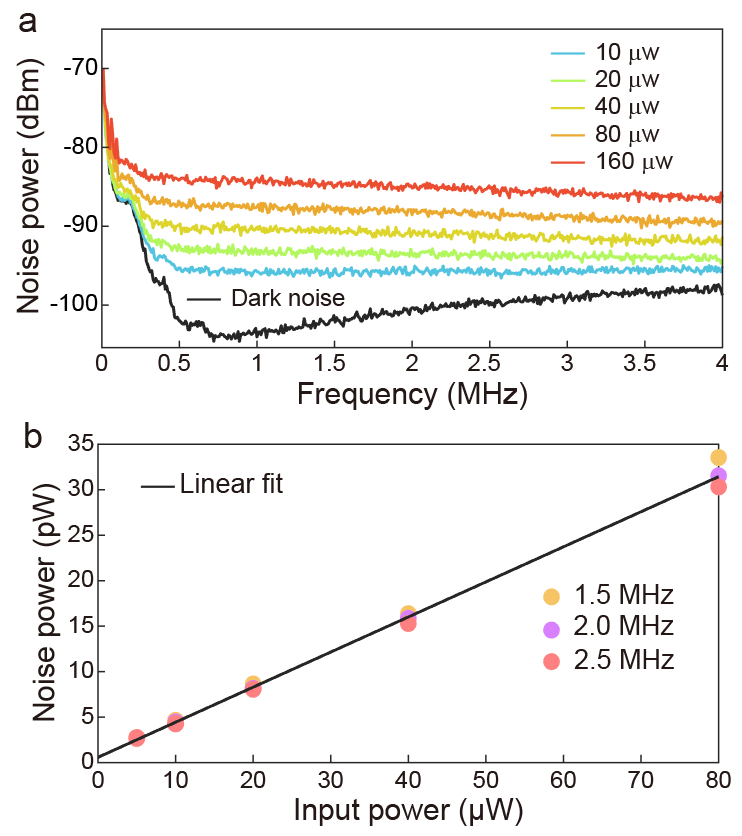


**Fig. S4** | **Characterization of balanced detection at the shot noise limit. a** Electrical spectra of the balanced outputs measured at different comb powers. All measurements are taken at 100 kHz resolution bandwidth. **b** Noise power dependence on incident comb power. The dark noise from the detector has been subtracted from the noise power. The linear trend indicates the balanced detector operates in the shot noise-limited regime [1].


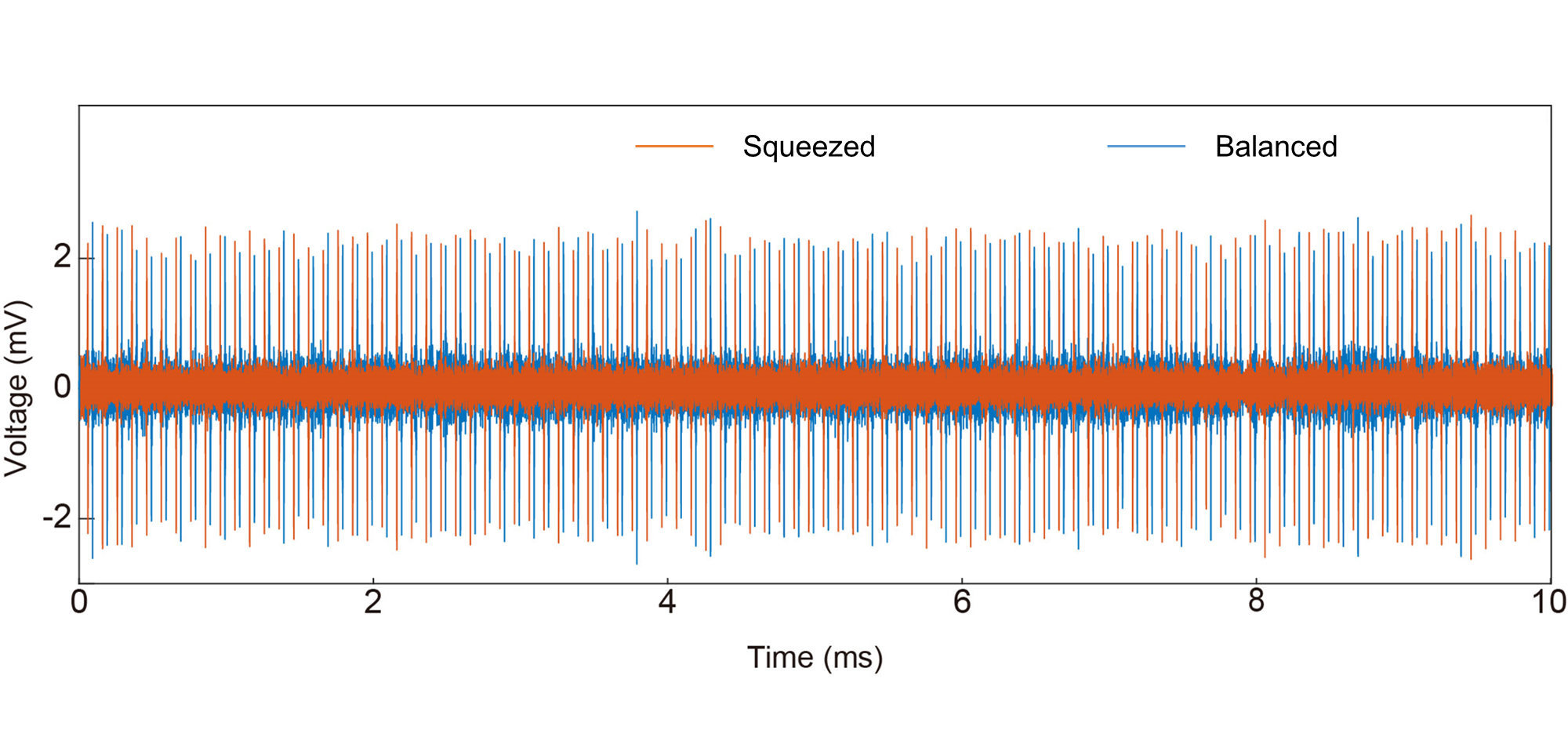


**Fig. S5** | **Time-domain dual-comb signals after bandpass filtering.** The data are mathematically filtered within the radio frequency range where the comb lines are present. The squeezed data (orange) are obviously less noisy than the balanced dual-comb data (blue).

Supplementary Table

**Table 1. Losses caused by light transmission and detection**

| Cause of loss | Loss (dB) | Transmission or detection efficiency |
| --- | --- | --- |
| Fiber facet reflection | 0.27 | 94% |
| Grating diffraction | 0.18 | 96% |
| Optical components  (mirrors, lenses, filters, PPLN crystal) | 0.46 | 90% |
| Photodetectors | 0.76 | 84% |
| Total | 1.67 | 68% |

Note that Supplementary Table 1 summarizes the optical components, showing a total loss of 0.46 dB for all mirrors and lenses, and an estimated loss of 0.2 dB for the up-conversion section (PPLN crystal, dichroic mirror 2, and parabolic mirror).

Supplementary Notes

**Note 1. Mathematical description**

The mathematical principles behind dual-comb spectroscopy (DCS) are well established in previous studies [2, 3]. In this work, we build upon those findings to clarify our experiment. Our scheme uses a signal comb that contains *N* spectral elements. The field operator for the signal comb can be expressed as follows [2]:

$$\hat{A}(t)=\frac{1}{\sqrt{T}}\left[\hat{a}(t)+\sum_{n=1}^{N} A_{n}\text{e}^{i2\pi nf_{r}t}\right]$$

where *T* is the integration time, *f*_r_ is the comb repetition frequency, and *A* represents the amplitude of a comb line. The total signal comb power is

$$P_{s}=hv_{0}\sum\left|A\right|^{2}\text{/}T$$

where *h* is Plank constant and *v*_0_ is the comb carrier frequency (*v*_0_>> *f*_r_). Correspondingly, the field operator for the local comb at a slightly different repetition frequency, *f*_r_ +Δ*f*, (Δ*f* << *f*_r_) is

$$\hat{B}(t)=\frac{1}{\sqrt{T}}\left[\hat{b}(t)+\sum_{n=1}^{N} B_{n}\text{e}^{i2\pi n(f+\text{Δ}f_{\text{r}})t}\right]$$

and the local comb power is

$$P_{lo}=hv_{0}\sum\left|B\right|^{2}\text{/}T$$

The two combs combine on a 50:50 beamsplitter, yielding two outputs:

$$\hat{d}_{\pm}(t)=[\hat{A}(t)\pm\hat{B}(t)]\text{/}\sqrt{2}$$

Two identical photodetectors detect the outputs for balanced detection, yielding photon counts

$$\hat{N}(t)=\hat{d}_{+}^{+}(t)\hat{d}_{\text{+}}(t)\text{-}\hat{d}_{-}^{+}(t)\hat{d}_{-}(t)=\hat{A}^{+}(t)\hat{B}(t)+\hat{B}^{+}(t)\hat{A}(t)$$

After passing through a gas cell, the signal comb field becomes

$$A_{n}\to\sqrt{\kappa_{n}}A_{n}\text{e}^{i\alpha_{n}}$$

with the transmissivity *κ*(*f*) and phase shift *α*(*f*) introduced by molecules. Consequently, the dual-comb beating signals within a radio-frequency range from 0 to *f*_r_/2 can be expressed as:

$$\bar{N}_{\text{DCS}}(t)=\frac{1}{T}\left[\sum_{n=1}^{N} \sqrt{\kappa_{n}}\text{e}^{i\alpha_{n}}A_{n}B_{n}\text{e}^{i2\pi n\text{Δ}f_{\text{r}}t}+\text{c}.\text{c}.\right]$$

where c.c. represents the complex conjugate. Here, transmission loss and detection efficiency are not taken into account, and the initial phase difference between the local and signal combs is zero, as it can be controlled in the experiments. Consequently, the mean of dual-comb beating signals in the Fourier-transform frequency domain is expressed as:

$$\bar{N}_{\text{DCS}}(m\text{Δ}f_{\text{r}})=\sqrt{\kappa_{m}}A_{m}B_{m}\text{e}^{i\alpha_{m}},1\leq m\leq N$$

In general, the complex noise characteristics in DCS can be categorized into two main groups [3]: additive noise and multiplicative noise. Additive noise includes factors such as comb relative intensity noise (RIN), detector noise defined as noise equivalent power (NEP), dynamic range limitations imposed by detectors and digitizers, and fundamental shot noise. On the other hand, multiplicative noise arises from relative timing and phase noise between the two combs. In practice, there is a specific power regime (e.g., μW to mW) where shot noise becomes the dominant factor. The origin of shot noise includes fluctuations that resemble quadrature-like behavior due to the interaction of coherent states from both the local comb and the paired coherent states from the signal comb at the beamsplitter. Additionally, intensity-like fluctuations can occur from the interaction of coherent states from each comb with a vacuum state at the beamsplitter. For dual-comb systems with small duty cycles, intensity-like fluctuations tend to be a significant source of noise. In such cases, the noise in DCS can be expressed as:

$$\begin{matrix} \text{var}[N_{\text{DCS}}(m\text{Δ}f_{\text{r}})]\text{=var}\left[\hat{\text{Σ}}_{\text{DCS}}(m\text{Δ}f_{\text{r}})\right]\approx\sum_{n=1}^{N} \kappa_{n}\left|A_{n}\right|^{2}+\left|B_{n}\right|^{2} \\ \text{ =}\kappa P_{s}T/hv_{0}+P_{lo}T/hv_{0} \end{matrix}$$

The shot-noise limited (SNL) signal-to-noise ratio (SNR) would be

$$\text{SNR}_{\text{SNL}}^{2}=|\bar{N}_{\text{DCS}}(m\text{Δ}f_{\text{r}})|^{2}/\text{var}[N_{\text{DCS}}(m\text{Δ}f_{\text{r}})]=\frac{T}{N^{2}hv_{0}}\frac{\kappa P_{s}P_{lo}}{\kappa P_{s}+P_{lo}}$$

In quantum correlation-enhanced DCS, we utilize a quantum correlated comb to mitigate the shot noise generated by the local comb. These quantum correlated combs are produced through seeded four-wave mixing (SFWM) in a highly nonlinear fiber. The optical field operators involved in SFWM are illustrated in Supplementary Fig. S1. According to the theoretical models of two-mode squeezing [4-6], the amplified seed and idler output fields are described as:

$$\begin{matrix} \hat{a}_{out}=\sqrt{G}\hat{a}_{0}+\sqrt{G-1}\hat{c}_{0}^{+} \\ \hat{c}_{out}=\sqrt{G-1}\hat{a}_{0}^{+}+\sqrt{G}\hat{c}_{0} \end{matrix}$$

The two output fields are detected by two identical photodetectors in a balanced configuration, producing a subtracted photocurrent (*i*) that has variance of

$$\mathrm{var}(i)=\mathrm{var}(\hat{a}_{out}^{+}\hat{a}_{out}-\hat{c}_{out}^{+}\hat{c}_{out})=\mathrm{var}(\hat{a}_{0}^{+}\hat{a}_{0})=<\hat{a}_{0}^{+}\hat{a}_{0}>=\frac{T}{hv_{0}}P_{lo}$$

where the local power is amplified to *G*·*P_lo_* and *G* represents the gain. When two identical signal fields, $\hat{b}_{s}$, are added for heterodyne detection, the noise term becomes

$$\begin{matrix} \text{var(}i\text{)}=\mathrm{var}((\hat{a}_{out}+\hat{b}_{s})^{+}(\hat{a}_{out}+\hat{b}_{s})-(\hat{c}_{out}+\hat{b}_{s}^{+})^{+}(\hat{c}_{out}+\hat{b}_{s}^{+})) \\ =<\hat{a}_{0}^{+}\hat{a}_{0}+2\hat{b}_{s}^{+}\hat{b}_{s}+(\sqrt{G}-\sqrt{G-1})^{2}\hat{a}_{0}^{+}\hat{a}_{0}+(\sqrt{G}-\sqrt{G-1})^{2}>. \end{matrix}$$

For *G*≫1, this noise term can be approximated as

$$\text{var}[N_{\text{AC}}(m\text{Δ}f_{\text{r}})]\text{=var(}i\text{)}\approx<\hat{a}_{0}^{+}\hat{a}_{0}+2\hat{b}_{s}^{+}\hat{b}_{s}>\text{=}(P_{lo}+2\kappa P_{s})\frac{T}{hv_{0}}$$

Consequently, the dual-comb SNR would be

$$\text{SNR}_{\text{QC-DCS}}^{2}=|\bar{N}_{\text{AC}}(m\text{Δ}f_{\text{r}})|^{2}/\text{var}[N_{\text{AC}}(m\text{Δ}f_{\text{r}})]=\frac{T}{N^{2}hv_{0}}\frac{\kappa P_{s}P_{lo}\cdot G}{2\kappa P_{s}+P_{lo}}$$

For *P*_lo_>>*P*_s_, the SNRs for the SNL and squeezed cases would be

$$\text{SNR}_{\text{SNL}}^{2}\approx\frac{T}{N^{2}hv_{0}}\frac{\kappa P_{s}}{P_{lo}}$$

$$\text{SNR}_{\text{QC-DCS}}^{2}\approx\frac{T}{N^{2}hv_{0}}\frac{\kappa P_{s}\cdot G}{P_{lo}}$$

Therefore, our approach ideally improves the SNR by a factor of √*G* as

$$\text{SNR}_{\text{QC-DCS}}=\sqrt{G}\cdot\text{SNR}_{\text{SNL}}$$

However, from a practical perspective, the improvement is limited by the efficiency of SFWM and the losses that occur during the squeezing and optical detection processes.

Notably, classical or non-quantum methods have been demonstrated to enhance the SNR of comb measurements. For instance, exploiting the pulsed nature of a comb, temporal gate filtering markedly enhances the SNR of heterodyne measurements between a comb and a continuous-wave (CW) laser (e.g., by a factor of 50). However, dual-comb interferometric signals obtained through multi-heterodyne detection with two combs exhibit intricate time-domain characteristics. Techniques like temporal gating may distort dual-comb spectra, thereby compromising precise measurements of molecular line positions and intensities. Alternatively, alias spectral averaging has improved the SNR of DCS by averaging signals from multiple dual-comb aliases. This approach necessitates substantial pulse chirping to minimize correlation among spectral replicas. However, this decorrelation process may impact precision measurements, warranting further experimental investigation.

The quantum-enhanced scheme we propose leverages quantum corrections to suppress shot noise, thereby boosting SNR beyond the quantum limit without forfeiting the advantages of DCS in molecular spectroscopy. Crucially, our scheme is not mutually exclusive with the aforementioned classical methods. While our approach centers on quantum-correlated comb sources, the classical methods focus on signal detection and data processing. These strategies can potentially collaborate to comprehensively enhance DCS capabilities.

**Note 2. Discussion on optical losses**

Optical losses due to light propagation and detection can be modeled by a beam splitter with an unused port. The output state is a combination of the input light and vacuum modes introduced through the empty port, which add vacuum fluctuations to the transmitted beam. The impact of such losses on intensity difference squeezing has been extensively studied [1, 4-6]. Here, we utilize the main conclusions. Ideally, the degree of squeezing can be expressed as *S*_0_=1/(2*G*-1) [6]. When affected by optical loss, it becomes

*S* = *η*·*S*_0_ + (1-*η*),

where *η* represents the transmission and detection efficiency, assuming identical efficiency for the correlated twin beams. Experimentally, we achieved a maximum squeezing of 3.5 dB. The theoretical value for squeezing, given by -10·lg(*S*), is 4.5 dB for *η*= 68% (Supplementary Table 1) and gain *G*=20. The discrepancy arises due to noise generated in the nonlinear fiber during SFWM [6]. By employing detectors with 98% quantum efficiency, the squeezing could improve to 6.5 dB. Besides, by using the above formula, one can estimate a generated squeezing (*S*_0_) at the fiber output, when knowing *η* and the measured degree of squeezing, *S*.

References

1. Yang, Z. J. et al. A squeezed quantum microcomb on a chip. *Nature Communications* **12**, 4781 (2021).
2. Shi, H. W. et al. Entanglement-enhanced dual-comb spectroscopy. *npj Quantum Information* **9**, 91 (2023).
3. Newbury, N., Coddington, I. & Swann, W. Sensitivity of coherent dual-comb spectroscopy. *Optics Express* **18**, 7929-7945 (2010).
4. Liu, S. S., Lou, Y. B. & Jing, J. T. Interference-induced quantum squeezing enhancement in a two-beam phase-sensitive amplifier. *Physical Review Letters* **123**, 113602 (2019).
5. Guo, X. S. et al. Multimode theory of pulsed-twin-beam generation using a high-gain fiber-optical parametric amplifier. *Physical Review A* **88**, 023841 (2013).
6. Jasperse, M., Turner, L. D. & Scholten, R. E. Relative intensity squeezing by four-wave mixing with loss: an analytic model and experimental diagnostic. *Optics Express* **19**, 3765-3774 (2011).
